# On the debated role of temporo-parietal dysfunction in patients with basal ganglia neglect

**DOI:** 10.3389/fnbeh.2013.00168

**Published:** 2013-11-18

**Authors:** Luca F. Ticini

**Affiliations:** The Italian Society for Neuroaesthetics ‘Semir Zeki’Trieste, Italy

**Keywords:** spatial neglect, visual extinction, perfusion-weighted imaging, temporoparietal junction, basal ganglia

A lively debate in neuropsychology addresses the question of which neural structure, when damaged, causes spatial neglect. Behaviorally, individuals with spatial neglect show several disturbances including the spontaneous deviation of head and eyes toward the ipsilesional (right) side as well as inattention to objects and persons located on the contralesional side of space (for a review, see Karnath and Rorden, 2012). Occasionally, neglect is associated with visual extinction, a deficit in the ability to simultaneously perceive multiple competing stimuli (Oppenheim, 1885). Neglect and extinction show obvious differences and several scholars have argued that they represent separate deficits (Bisiach, 1991; Vallar et al., 1994; Driver et al., 1997; Milner, 1997; Karnath and Rorden, 2012). Moreover, they seem to have anatomically related but (partly) separated neural representations (Karnath et al., 2003). In particular, spatial neglect is provoked by damage to a spatially distributed right hemisphere network of cortical and subcortical structures that directly or remotely impairs brain areas dedicated to the orientation of visuospatial attention (e.g., Karnath et al., 2009; Thiebaut de Schotten et al., 2012; for a recent meta-analysis, see Molenberghs et al., 2012 and comments in Bartolomeo, 2012). Extinction, on the other hand, occurs when the right temporo-parietal cortex is directly damaged (Karnath et al., 2003; see also Molenberghs et al., 2012, Figure 3) or experimentally deactivated by transcranial magnetic stimulation (Meister et al., 2006).

Subcortical lesions can also lead to neglect (Demeurisse et al., 1997; Hillis et al., 2005) and extinction (Ticini et al., 2010a) when they compromise the functioning of districts located far from the neural loss itself, through pathophysiological mechanisms such as hypometabolism (Yasaka et al., 1998), hypoperfusion (Hillis et al., 2000), or diaschisis (Monakow, 1914; Kempler et al., 1988; Price et al., 2001; Finger et al., 2004). Indeed, at times, neglect and extinction are associated with areas of dysfunctional but structurally intact tissue beyond the structural damage itself (e.g., Hillis et al., 2001, 2005; Karnath et al., 2005; Ticini et al., 2010a; Khurshid et al., 2012). How these brain regions are identified?

In the acute stage of a stroke, Diffusion-Weighted MR Imaging (or T2-weighted fluid-attenuated inversion-recovery sequences if imaging is conducted 48 h or later after stroke onset) is used to reveal the irreversibly damaged areas (Fisher, 1995). Perfusion-Weighted Imaging (PWI) is employed, instead, to identify the whole extent of dysfunctional tissue within and beyond the lesion. The mismatch between diffusion and perfusion maps (“diffusion-perfusion mismatch”; see Neumann-Haefelin et al., 1999) spots areas of functional deficits besides the structural damage (e.g., the tissue that receives enough blood supply to remain structurally intact but not enough nourishment and oxygen to function normally) that are potentially recoverable if blood flow is restored by medical intervention (Beaulieu et al., 1999). As a matter of fact, recent results have shown that restoring blood flow ameliorates different types of neglect, thus suggesting the factual role of dysfunctional brain areas in this syndrome (Khurshid et al., 2012). Nonetheless, some uncertainties still endure. In the following lines I aim at arguing against the role of the temporo-parietal junction in subcortical neglect, suggesting instead that this area is dysfunctional in extinction, in conformity with previous studies (e.g., Karnath et al., 2003; Meister et al., 2006).

In a paper published in the journal *Brain*, Karnath et al. (2005) employed spatial normalization and symmetric voxel-wise inter-hemispheric comparisons, to precisely locate the functional deficits associated with neglect in patients with subcortical sub/acute lesions. The authors found that: “strokes centering on the right basal ganglia which provoke spatial neglect induce abnormal perfusion in a circumscribed area of intact cortex that typically involves those three regions that have previously been described to provoke spatial neglect when damaged directly by cortical infarction: the superior temporal gyrus, the inferior parietal lobule and the inferior frontal gyrus” (i.e., the inferior frontal and temporo-parietal cortices; see their Figure 3). They further concluded that: “spatial neglect following a right basal ganglia lesion typically is caused by the dysfunction of (part of) these specific cortical areas.” To investigate the abnormally perfused tissue in basal ganglia neglect, Karnath et al. (2005) “compared perfusion abnormalities in the patient groups with and without spatial neglect.” For this purpose, they subtracted the diffusion-perfusion mismatch maps of 5 control patients *without* neglect from those of 5 individuals *with* neglect.

It is worth noting at this point that to identify brain-behavior correlations in stroke patients, it is common to employ the well-established procedure that consists in subtracting the images of the group without neurological or neuro-psychological symptoms from the images of the group who has those symptoms (Rorden and Karnath, 2004). Evidently, a critical prerequisite for the successful application of this technique is the selection of appropriate patient groups. However, in Karnath et al. the groups studied were not selected according to this procedure: indeed, 4 of the neglect patients *also* had visual extinction. As such, they were later used by Ticini et al. (2010a) who subtracted diffusion-perfusion mismatch maps of 5 patients with *only* neglect from those of 8 individuals with neglect *and* extinction to find that visual extinction following a right basal ganglia lesion is caused by the dysfunction of the temporo-parietal area (for its involvement in extinction, see also de Haan et al., 2012). Noteworthy, in Ticini et al. (2010b) patients E1, E2, E4, and E5 (see their Table 1)—who belonged to the group with neglect *and* extinction—and patients N4—who belonged to the group with *only* neglect—were the 5 individuals “*with* neglect” studied by Karnath et al. (2005). In other words, there is the possibility that, by subtracting the perfusion maps of control patients from that of the 5 neglect individuals of which 4 also had extinction, Karnath et al. overestimated the dysfunctional area associated with neglect, by extending it to the temporo-parietal cortex in reality associated with extinction. The fact that patient N4 (i.e., the only neglect patient *without* extinction) “did not show relevant cortical perfusion abnormalities in addition to her lesion” (Karnath et al., 2005) supports this hypothesis.

In conclusion, these results juxtaposed with each other seem to suggest that visual extinction (rather than spatial neglect) following a sub/acute lesion in the right basal ganglia is caused by the dysfunction of the temporo-parietal cortex. The generalization of these results is limited by the small group size and the absence of information about spontaneous recovery at different stages after stroke-onset (as for instance in Khurshid et al., 2012). Nonetheless, the information provided here may contribute to an incremental understanding of the role of perfusion deficits in spatial neglect and extinction through a better characterization of potentially salvageable brain tissue associated with these neuropsychological deficits.
